# Phase Structure and Magnetic Properties of Nanocrystalline ThMn_12_-Type (Nd_1−x_Sm_x_)_1.2_Fe_10.5_Mo_1.5_ Alloys

**DOI:** 10.3390/ma19050930

**Published:** 2026-02-28

**Authors:** Weiwei Zeng, Xiao He, Bohe Luan, Shanshan Ren, Xuefeng Liao, Qing Zhou

**Affiliations:** 1 Guangdong Provincial Key Laboratory of Rare Earth Development and Application, Institute of Resources Utilization and Rare Earth Development, Guangdong Academy of Sciences, Guangzhou 510640, China; zengweiwei@grre.gd.cn (W.Z.); 18279193068@163.com (B.L.); liaoxuefeng@grre.gd.cn (X.L.); 2 Institute of Rare Earth Permanent Magnets, Heyuan Rising-Guangdong Academy of Sciences, Heyuan 517000, China; 3 Faculty of Metallurgical and Energy Engineering, Kunming University of Science and Technology, Kunming 650093, China; renshanshan@ymu.edu.cn

**Keywords:** ThMn_12_ structure, Nd-based, coercivity, magnetocrystalline anisotropy field

## Abstract

Nd-based ThMn_12_ alloys exhibit significant potential as rare-earth (RE)-lean permanent magnets; however, their reliance on a nitriding process imposes limitations on densification due to the thermal instability of nitrides. Herein, we investigate the substitution of Nd with Sm in nanocrystalline melt-spun (Nd_1−x_Sm_x_)_1.2_Fe_10.5_Mo_1.5_ alloys to enhance magnetic performance without nitrogenation. The results confirm that Sm substitution preserves the tetragonal ThMn_12_-type phase as the dominant matrix across all alloys, ensuring structural stability. Magnetic measurements demonstrate a significant enhancement in both coercivity *µ*_0_*H*_c_ and remanence *µ*_0_*M*_r_, attributed to the strengthened magnetocrystalline anisotropy and improved squareness of the demagnetization curves induced by Sm substitution. Furthermore, microstructural characterization indicates that Sm facilitates the preferential formation of the REFe_7_ phase under identical rapid solidification conditions. This work provides a strategic pathway to tailoring the magnetic properties of Nd-based ThMn_12_ alloys, rendering them capable of exhibiting permanent magnet behavior without nitrogenation.

## 1. Introduction

The growing global demand for permanent magnets in clean energy technologies and electric mobility has intensified the urgency to reduce reliance on rare-earth (RE) elements, driven by concerns over resource scarcity and environmental sustainability [1]. The tetragonal RE(Fe,M)_12_ compounds (M = stabilizing transition metal), which feature the ThMn_12_ structure, have emerged as promising candidates due to their reduced RE content of ~7.7 at.% compared to conventional Nd_2_Fe_14_B magnets (~11.8 at.%) [2,3,4]. Over the past decade, research has focused predominantly on Sm-based and Nd-based ThMn_12_ compounds. Sm(Fe,M)_12_ systems, in particular, have demonstrated remarkable progress through diverse processing routes such as coating, melt-spinning [5,6,7,8,9,10], bonding [11,12], hot-deformation [13,14] and sintering [15,16,17]. Notably, sintered Sm_8_Fe_73.5_Ti_8_V_8_Ga_0.5_Al magnets have achieved coercivity *μ*_0_*H*_c_ over 1.0 T [18], positioning them as viable candidates for applications including drive systems, voice coil motors (VCMs), and magnetic resonance imaging (MRI) systems.

In contrast, Nd-based ThMn_12_ compounds have received less attention due to their inherently low magnetocrystalline anisotropy field (*µ*_0_*H*_A_) at room temperature [19,20]. This limitation arises from the dominance of the Fe sublattice in determining anisotropy, which results in typical *µ*_0_*H*_A_ values below 1.0 T for Nd(Fe,M)_12_ compounds. To enhance the anisotropy of Nd(Fe,M)_12_ compounds, interstitial nitrogenation has been explored as a promising strategy. Nitrogen incorporation elevates *µ*_0_*H*_A_ to 8.0–11.0 T by shifting anisotropy dominance to the Nd sublattice, thereby inducing strong uniaxial magnetic behavior [21,22,23]. However, this approach presents challenges, as the Nd(Fe,M)_12_N_x_ phases exhibit a decomposition process above 600 °C, hindering their consolidation into bulk magnets via sintering or hot pressing [24].

An alternative pathway to achieve high uniaxial anisotropy in Nd(Fe,M)_12_ systems involves the partial substitution of Nd with another RE element. Sm emerges as a compelling candidate due to the intrinsically higher *µ*_0_*H*_A_ of Sm(Fe,M)_12_ compounds [3,25,26,27]. Despite the lower crustal abundance of Sm compared to Nd, its current market price is much cheaper than that of Nd due to its limited industrial applications [28]. Capitalizing on this cost–performance balance, we synthesized a series of (Nd_1−x_Sm_x_)_1.2_Fe_10.5_Mo_1.5_ alloys via rapid quenching. This study systematically investigates the phase composition, microstructure, and magnetic properties of these alloys, aiming to establish foundational insights for advancing ThMn12-type magnet development.

## 2. Experimental Section

Ingots with nominal compositions of (Nd_1−x_Sm_x_)_1.2_Fe_10.5_Mo_1.5_ (x = 0, 0.2, 0.4, 0.6, 0.8 and 1.0) were prepared by arc-melting using 99.9% pure elements under Ar atmosphere. The ingots were directly quenched into nanocrystalline melt-spun ribbons by single-roller melt-spinning. Based on the process optimization results [7], a wheel speed of 15 m/s was selected to produce alloys with a nanocrystalline structure and a dominant ThMn_12_ phase. The resultant ribbon thickness, which ranged from approximately 10 to 30 µm, was controlled by adjusting these melt-spinning parameters. The phase constitutions of the samples in powder form were characterized by the X-ray diffractometer (X’ Pert Pro, PANalytical, Almelo, The Netherlands) with Cu-Kα radiation (λ = 1.5418 Å, 40 kV, 40 mA). The magnetic properties were obtained from a vibrating sample magnetometer (VSM) in the physical property measurement system (PPMS-9, Quantum Design, San Diego, CA, USA) with a maximum magnetic field of 5 T. For magnetic measurements, the melt-spun ribbons were cut into small pieces with a length of ~5 mm and width of ~2 mm. In-plane measurements were performed to minimize shape demagnetization effects, which are negligible for thin ribbons measured in-plane. The microstructure was examined with a scanning transmission electron microscope (Talos F200, FEI, Hillsboro, OR, USA) equipped with an energy dispersive spectrometer (EDS) and the specimens for TEM observation were prepared by ion milling (691, Gatan, Pleasanton, CA, USA).

## 3. Results and Discussion

**Figure 1:**
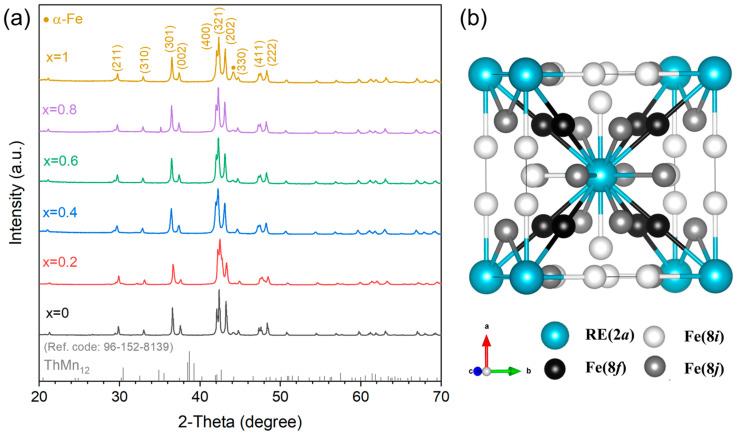
(**a**) The XRD patterns of melt-spun (Nd_1−x_Sm_x_)_1.2_Fe_10.5_Mo_1.5_ alloys and (**b**) schematic crystal structure of the ThMn_12_-type phase.

Figure 1a presents the XRD patterns of nanostructured melt-spun (Nd_1−x_Sm_x_)_1.2_Fe_10.5_Mo_1.5_ (x = 0, 0.2, 0.4, 0.6, 0.8 and 1.0) alloys. All samples are predominantly composed of the tetragonal ThMn_12_-type structure (I4/mmm) (Figure 1b), with minor traces of the cubic *α*-Fe phase. This observation demonstrates that the partial substitution of Nd by Sm does not significantly alter the primary phase composition of the alloys, a finding consistent with previous research [25]. The stability of the ThMn_12_-type structure is known to depend critically on the presence of stabilizing transition metal elements. In this system, Mo serves as the stabilizing element, and the incorporation of 1.5 at.% Mo is sufficient to maintain the structural integrity of the ThMn_12_ phase.

**Figure 2:**
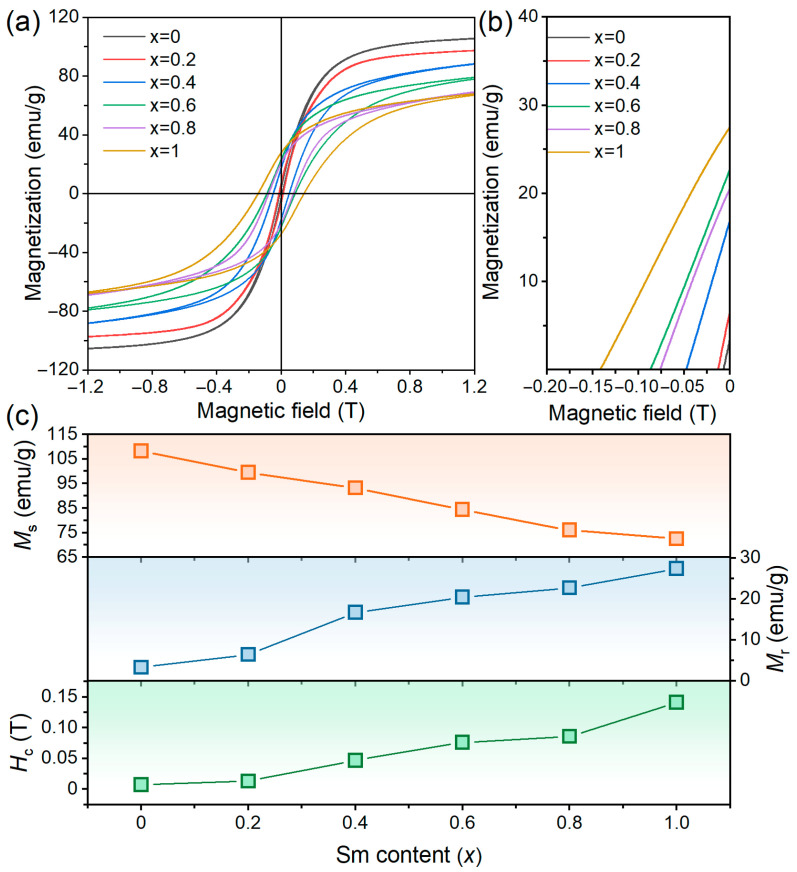
(**a**) Hysteresis loops, (**b**) demagnetization curves and (**c**) corresponding magnetic parameters of melt-spun (Nd_1−x_Sm_x_)_1.2_Fe_10.5_Mo_1.5_ alloys.

The room-temperature hysteresis loops of the melt-spun (Nd_1−x_Sm_x_)_1.2_Fe_10.5_Mo_1.5_ alloys are presented in Figure 2a. A systematic evolution in loop morphology is observed with increasing Sm substitution, in which the initially high and narrow profile transforms progressively into a shorter and wider configuration. This evolution reflects a significant enhancement in coercivity (*µ*_0_*H*_c_) accompanied by a reduction in saturation magnetization (*µ*_0_*M*_s_). Specifically, the Nd_1.2_Fe_10.5_Mo_1.5_ (x = 0) alloy exhibits a near-zero coercivity of 0.01 T, which can be attributed to its relatively low magnetocrystalline anisotropy field (*µ*_0_*H*_A_). The *µ*_0_*H*_c_ increases linearly with increasing Sm content, reaching 0.14 T for the fully Sm-substituted (x = 1.0) alloy. In this work, the achieved coercivity of 0.14 T for the Sm-rich (x = 1.0) alloy, obtained without nitrogenation, represents a significant improvement over the Nd-rich base alloy. While this value is lower than the ~0.57 T reported for early nitrided Nd(Fe,Mo)_12_N_x_ isotropic powders [4], it successfully circumvents the thermal instability inherent to nitride phases. Compared to contemporary Sm-based 1:12 systems processed via advanced powder metallurgy routes which can achieve *H*_c_ > 1.0 T in bulk consolidated forms [13], the present results highlight the potential of the Sm-substitution approach while also indicating that further gains in extrinsic properties may be realized through optimized microstructural control, such as grain refinement and the engineering of continuous, non-ferromagnetic grain boundary phases.

Intriguingly, despite the decline in *µ*_0_*M*_s_ with Sm substitution, the remanence (*µ*_0_*M*_r_) exhibits an inverse trend, as presented in Figure 2c. This apparent contradiction is rationalized by the improved squareness of the demagnetization curves, which arises from the increased *µ*_0_*H*_c_. Higher coercivity suppresses irreversible magnetization reversal during the demagnetization process, thereby enhancing *µ*_0_*M*_r_. These findings underscore that Sm substitution can effectively tailor the magnetic properties of Nd-based ThMn_12_ alloys, rendering them capable of exhibiting improved hard magnetic properties without a nitridation process.

**Figure 3:**
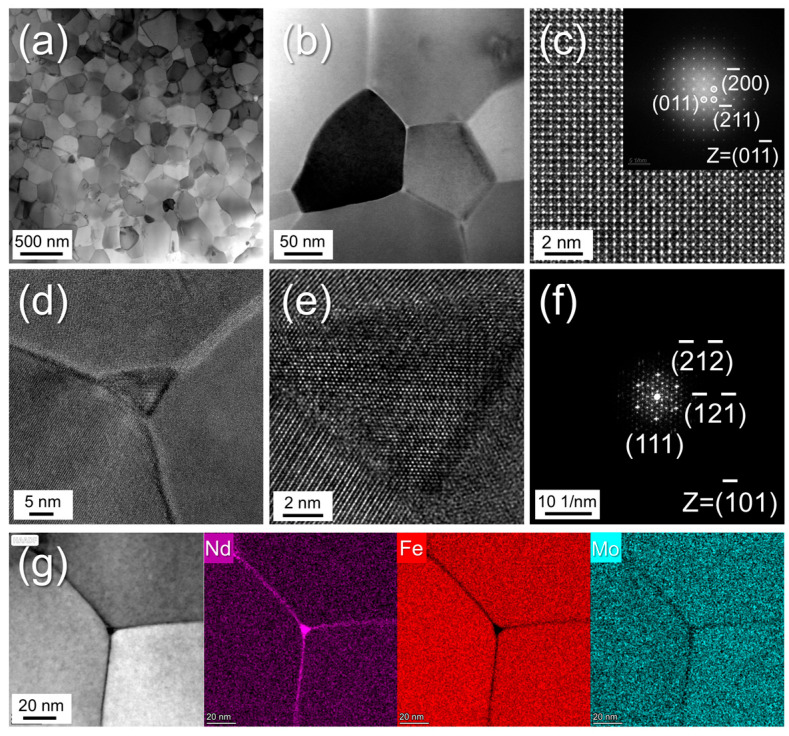
(**a**,**b**) Bright-field TEM images of melt-spun Nd_1.2_Fe_10.5_Mo_1.5_ (x = 0) alloy. High-resolution TEM images of selected (**c**) main grains and (**d**,**e**) typical triple junction regions. The fast Fourier transform (FFT) patterns obtained from (**c**) and (**e**) are shown in (**c**)’s inset and (**f**), respectively. (**g**) EDS elemental mapping of the typical triple junction regions.

Figure 3 illustrates the microstructure and elemental distribution of the melt-spun Nd_1.2_Fe_10.5_Mo_1.5_ (x = 0) alloy, characterized in the absence of Sm substitution. As depicted in Figure 3a,b, the alloy comprises well-crystallized equiaxed grains with an average size of ~146 nm. According to previous studies, the single-domain critical size (*D*_c_) for ThMn_12_-type compounds ranges from 150 to 300 nm, depending on the rare-earth (RE) and stabilizing elements [5,6]. Since the average grain size in the present sample is significantly below this single-domain threshold, the microstructure is favorable for achieving high coercivity. This observation aligns with the broader principle, evidenced in other low-dimensional magnetic systems [29], that the controlled reduction in grain size is a critical pathway for enhancing magnetic performance. High-resolution TEM analysis of a representative grain is depicted in Figure 3c. Energy-dispersive X-ray spectroscopy (EDS) reveals an atomic ratio of Nd to (Fe + Mo) of approximately 1:12 within the main phase, consistent with the ThMn_12_-type stoichiometry. This structural assignment is further corroborated by the fast Fourier transform (FFT) pattern shown in the inset of Figure 3c. Figure 3d focuses on the grain boundary regions, revealing two distinct features: (i) ultrathin intergranular boundary phases approximately 1–2 nm in thickness that separate adjacent ThMn_12_-type grains, and (ii) triple junction grain boundary regions. Elemental mapping of a typical triple junction, shown in Figure 3g, indicates significant Nd enrichment accompanied by Fe depletion within both the lamellar grain boundaries and triple junctions. In combination with the FFT analysis in Figure 3f, these results confirm that the triple junction phase corresponds to a hexagonal close-packed (*hcp*) Nd phase. The presence of this *hcp*-Nd grain boundary phase is critical for enhancing coercivity by effectively isolating magnetic coupling between adjacent ThMn_12_ grains. Furthermore, Mo is found to predominantly segregate within the ThMn_12_ matrix grains, consistent with its role as a stabilizing element for the ThMn_12_ structure.

**Figure 4:**
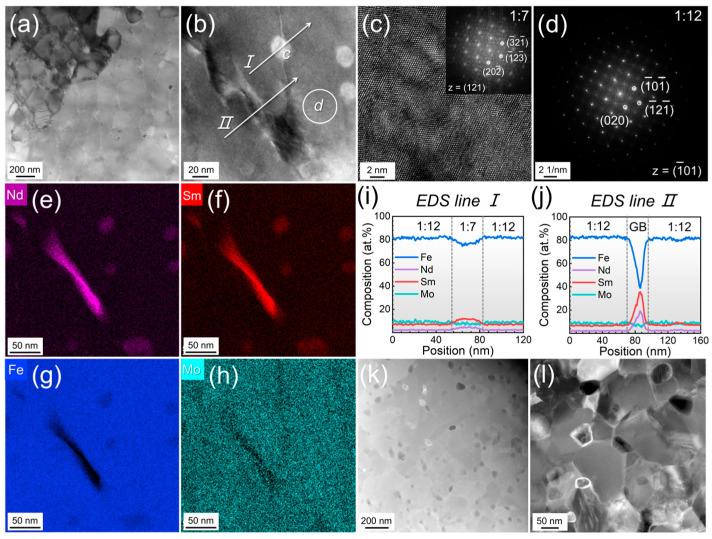
(**a**,**b**) Bright-field and (**c**) high-resolution TEM images of melt-spun (Nd_0.2_Sm_0.8_)_1.2_Fe_10.5_Mo_1.5_ (x = 0.8) alloy. The fast Fourier transform (FFT) patterns obtained from (**c**) and (**b**) are shown in (**c**)’s inset and (**d**), respectively. (**e**–**h**) EDS elemental mapping and (**i**,**j**) line scanning of (**b**). (**k**,**l**) Bright-field TEM images of melt-spun Sm_1.2_Fe_10.5_Mo_1.5_ (x = 1.0) alloy.

To further investigate the influence of Sm substitution on the microstructural evolution of the alloys, the microstructure of Sm-substituted alloys is analyzed in Figure 4. As illustrated in Figure 4a, the (Nd_0.2_Sm_0.8_)_1.2_Fe_10.5_Mo_1.5_ (x = 0.8) alloy exhibits an equiaxed grain structure with a grain size of ~138 nm, analogous to the Sm-free (x = 0) alloy. Notably, secondary phase particles with an average size of ~20 nm are observed both within the grain interiors and at grain boundaries, as highlighted in Figure 4b. The FFT analysis of the matrix grains and secondary phase particles, presented in Figure 4c and d, respectively, confirms that the matrix corresponds to the REFe_12_ (1:12) phase, while the secondary particles are identified as the REFe_7_ (1:7) phase. The 1:7 phase is a metastable phase, which is ferromagnetic. In contrast to non-ferromagnetic grain boundary phases, its presence at the interfaces of the main grains promotes magnetic exchange coupling across the boundaries, which is detrimental to coercivity. To elucidate elemental distribution, EDS elemental mapping, in Figure 4e–h, and line scanning, in Figure 4i,j, acquired from Figure 4b, were conducted. The results reveal that the grain boundary phase is enriched in Nd and Sm but depleted in Fe and Mo, whereas the 1:12 matrix grains are Fe-rich and deficient in Nd and Sm. The notably low Fe concentration within the grain boundary phase suggests that this region is likely non-ferromagnetic. Consequently, this phase aids in the magnetic decoupling of the main grains, which is beneficial for enhancing coercivity. Figure 4i further demonstrates that the 1:7 phase contains significantly higher concentrations of Sm and Nd compared to the 1:12 phase. The fully Sm-substituted Sm_1.2_Fe_10.5_Mo_1.5_ (x = 1.0) alloy, shown in Figure 4k,l, displays a similar microstructure to the partially substituted alloys, with 1:7 phase particles precipitating within the 1:12 matrix grains and along grain boundaries. Collectively, these findings indicate that the introduction of Sm into the Nd(Fe,Mo)_12_ system promotes the preferential formation of the 1:7 phase under identical rapid solidification conditions.

## 4. Conclusions

In this work, nanocrystalline melt-spun (Nd_1−x_Sm_x_)_1.2_Fe_10.5_Mo_1.5_ alloys with Sm substitution levels spanning x = 0 to 1 were successfully synthesized and systematically characterized for their structural and magnetic properties. Sm substitution does not compromise the structural stability of the tetragonal ThMn_12_-type phase, which dominates the alloy matrix across all compositions. A notable enhancement in both coercivity *µ*_0_*H*_c_, from 0.01 T (x = 0) to 0.14 T (x = 1.0), and remanence *µ*_0_*M*_r_, from 0.29 T to 0.35 T, is observed with increasing Sm substitution, which is indicative of strengthened magnetocrystalline anisotropy and squareness of the demagnetization curves due to Sm incorporation. Microstructural characterization reveals that Sm substitution facilitates the preferential formation of the 1:7 phase under identical rapid solidification conditions. Overall, this work provides a promising strategy for enhancing the magnetic properties of Nd-based ThMn_12_ alloys without the need for nitrogenation.

## Data Availability

The original contributions presented in this study are included in the article. Further inquiries can be directed to the corresponding authors.
